# Vision and Relevant Risk Factor Interventions for Preventing Falls among Older People: A Network Meta-analysis

**DOI:** 10.1038/srep10559

**Published:** 2015-05-28

**Authors:** Xin-Yi Zhang, Jian Shuai, Li-Ping Li

**Affiliations:** 1 Injury Prevention Research Center, Medical College of Shantou University, Shantou, Guangdong, China

## Abstract

Our study objective was to determine the effect of vision intervention and combinations of different intervention components on preventing falls and fall-related injuries among older people. Six electronic databases were searched to identify seven articles published before May, 2014. We conducted a systematic review of data from seven randomized controlled trails and identified eight regimens: vision intervention alone (V), vision plus exercise (referred to as physical exercise) interventions (V + E), vision plus home hazard interventions (V + HH), vision plus exercise plus home hazard interventions (V + E + HH), vision plus exercise plus sensation interventions (V + E + S), vision plus hearing interventions (V + H), vision plus various risk factor assessment and interventions (V + VRF), and the control group (C, no intervention group). The main outcome was the incidence of falls during the follow-up period. Seven papers included 2723 participants. Network meta-analysis of seven trials, using pairwise comparisons between each intervention, indicated there was no significant difference. However, there was a trend in which intervention incorporating V + VRF had more advantages than any other combination of interventions. In conclusion, V + VRF proves to be more effective than other V combination interventions in preventing falls in older people (≥65 years of age). V alone appears less effective in our network meta-analysis.

Falling is a common problem for senior citizens, with a third of those 65 years of age, and half of those over 85 years of age, falling each year^1^. Falls lead to moderate to severe injuries, fear of falling, loss of independence and death in a third of those patients^2^. In 2012, the direct medical cost of falls by the elderly, adjusted for inflation, was $30 billion^3^. With the population aging, both the number of falls and the cost to treat injuries due to falls are likely to increase. By 2020, the annual direct and indirect cost of fall injures is expected to reach $67.7 billion (in 2012 dollars)^4^.

In order to reduce the cases for fall injuries and huge medical expenses, many kinds of interventions to prevent falls of senior citizens, including improving vision, strengthening exercise, and managing home hazards, have been tried. Compared to exercise and home hazard management, it is more difficult to implement vision intervention in a population with limited access to eye care professionals. Currently, actual rates of eye examinations for persons diagnosed with the study conditions fall far short of recommended rates^5^. So there is little research of this kind and the ability of vision intervention to prevent falling remains unknown.

The prevalence of vision impairment and blindness increases with age^6^. Vision loss is a risk factor for falls of older people, and also is one of the most easily overlooked risk factor because the process of decreasing vision is slow and unnoticeable in most of the elderly. Several studies have found that impaired visual acuity increases the risk of falls^7^,^8^ and injuries^9^. Bilateral visual field (VF) loss from glaucoma is associated with greater fear of falling, with an impact that exceeds numerous other risk factors^10^. In a study among elderly women who lose visual acuity, the authors concluded loss of vision increases the risk of frequent falls, and prevention or correction of visual loss may help reduce the number of future falls^7^. Improving visual function may have benefits, such as decreased traumatic events and improved mobility^11^. However, randomized controlled trials suggest that this is often, but not always true^12^. In fact, well-meaning interventions can increase the risk of falls, so changes should be made with care. In orthopedic research on elderly falling, researchers found that the falling rate of the visually intervened group could be higher than that of the control group^13^.

The pathogenesis of falling is multifactorial^14^. Falling can result from impairments in balance, gait, muscle strength, visual acuity and cognition, chronic diseases and use of psychotropic medication^15^,^16^,^17^,^18^,^19^. A meta-analysis showed that a multifactorial fall risk assessment and management program was effective in all older populations investigated, both with high or low risk of falling^20^. However, it has not yet been demonstrated that multiple or multifactorial interventions prevent more falls than single, targeted interventions. In a recently completed trial of two interventions we showed that intervention was more effective when delivered singly, preventing more falls, than in combination^21^.

Whether vision intervention is effective for preventing falls is uncertain. Similarly, it remains unclear whether single intervention will be more effective than in combination with other interventions. We utilized network meta-analysis in order to make sure whether vision intervention alone and in combination with interventions for related risk factors is effective for preventing elderly falls.

## Research Methods

### Search strategy

A thorough review was carried out on six electronic databases, including EMBASE (1966 to May 4, 2014), PubMed (1950 to May 4, 2014), the Cochrane library (1800 to May 4, 2014), Web of Science (1900 to May 4, 2014), Medline (-to May 4, 2014), and CBM (-to May 4, 2014). These databases were searched comprehensively using the subject terms [(vision or visual or ocular) and (accidental falls or falls or fall or falling) and (aged or aging or elder care or elderly or elderly care or geriatric or geriatric assessment or older or senior) and (controlled clinical trial or randomized controlled trail or randomized or placebo or randomly or trial or groups)].

### Study selection

There were no limits placed on the year of publication. Studies were included if they (i) involved an RCT (randomized controlled trial), were blinded or not, with a follow-up period, (ii) involved a vision intervention, regardless of single or multiple interventions, (iii) regularly recorded a fall case use card, (iv) involved study subjects older than 65 years of age, and (v) were in English. We excluded studies with (i) less than 6 months of follow-up time, (ii) no specific interventions of ophthalmology, (iii) self-reported instead of actual eye examinations, and (iv) results of interest not or partially reported, e.g. studies with results that were not falls. Two reviewers independently screened all titles, abstracts and full texts and applied the inclusion and exclusion criteria. In a series of publications of a specific result, we routinely choose the latest one.

### Data extraction

Data on the first author, year of publication, location of the population, research period and affiliated institution, follow-up length, participant characteristics, falling rate, ophthalmologic examination and vision intervention classification were extracted by two authors (Zhang and Shuai) independently.

### Quality evaluation

The Cochrane Collaboration risk of bias tools were used to assess the risk of bias as follows: (i) random sequence generation, (ii) allocation concealment, (iii) blinding, (iv) incomplete outcome data, (v) selective outcome reporting, (vi) other potential biases (Table 1). We assessed recall bias by examining the time period over which fall recall occurred. It has been reported that fall recall over a three month period is inaccurate^22^ and that falls should be reported no less frequently than monthly^12^. Discrepancies between the two reviewers were arbitrated by a third reviewer (Li). Falling rates were quantified using the odds ratio (OR) and 95% confidence interval (CI).

A fall was defined as an unintentional change in position resulting in coming to rest at a lower level or on the ground^23^. A fall injury was considered present when the incident resulted in a head injury or a fracture or wound.

### Interventions

Vision intervention (V) included all measures that can improve vision, e.g. ophthalmologic examination, prescription glasses and cataract surgery. Exercise intervention (E) involved individualized exercises and group exercises aimed at improving flexibility, leg strength, balance and coordination. Sensation intervention (S) was to compensate for reduced peripheral sensation, e.g. use of a walking stick or wearing shoes with low heels. Hearing intervention (H) utilized a hearing aid and hearing remedies to improve sensory functions. Home hazard intervention (HH) provided free labour and materials to modify the home, e.g. modifications to floor coverings, fitting of hand rails, and maintenance to steps or ramps. Various risk factor (VRF) assessments included fall risk factor (blood pressure, medication review and lighting level) assessment, and intervention included modification of these risk factors and a 30-min group exercise session to music twice weekly. Our multiple component interventions consisted of a fixed combination of vision intervention plus one or two interventions mentioned above, e.g. vision plus exercise intervention (V + E) or vision plus home hazard intervention (V + HH). Vision intervention (V) was included in each multiple component intervention in our meta analysis. There were seven types of intervention groups in our study, including single vision intervention (V) or vision plus other risk factor interventions. Two studies had two or more intervention arms, some of them comprising vision plus multiple component interventions, and were separately compared with the control arm^24^,^25^. Two studies had vision plus one other risk factor intervention^13^,^26^. Three studies had single vision intervention^27^,^28^,^29^.

### Statistical analysis

We grouped regimens into vision intervention (V), vision plus exercise interventions (V + E), vision plus home hazard interventions (V + HH), vision plus exercise plus home hazard interventions (V + E + HH), vision plus exercise plus sensation interventions (V + E + S), vision plus hearing interventions (V + H), vision plus various risk factor assessments and interventions (V + VRF), and the control group (C).

### Traditional meta-analysis

A standard pair-wise meta-analysis was performed with a random effect model to conduct the vision intervention comparisons (V and C). This study was analyzed by the intention-to-treat. Every participant who was randomized in the seven reports was considered to be part of the study no matter if he or she completed the trial. We conducted the missing data by substituting conservative means and input outcomes^30^. Some outcome variables regarding the number of falls were estimated according to the fall incidence ratio. The OR and 95% CI were used to summarize statistics. I^2^ statistics were used to assess the heterogeneity of the meta-analysis. As a judgment standard, I^2^ with values >50% indicated high, 25–50% moderate, and <25% low heterogeneity^31^. Traditional meta-analysis was performed with STATA software (version 12.0; Statacorp, College Station, TX, USA).

### Network meta-analysis

A Bayesian framework of network meta-analysis was used to take all evidence into account simultaneously (both direct and indirect comparisons)^32^. OR (95%CI) and probability of being best of any given intervention with a random effect model. Sampling of posterior probabilities was performed by MCMC (Markov Chain Monte Carlo) method^33^. Each chain used 30,000 iterations with a burn in of 10,000. Non-informative priors were used throughout. We used the posterior mean of the residual deviance and deviance information criteria to assess the model’s goodness of fit^34^. A model is considered as a good fit when the residual deviance was close to the number of data points^35^. Moreover, we evaluated the probability that every intervention was best in terms of efficacy, and second, up to eighth best^34^. The OR was calculated when every intervention group made comparison with the control group. Our network meta-analysis was performed with the R2WinBUGS (version 2.1-19) package of R software (version 3.0.3; Development Core Team, http://www.r-project.org/) and “WinBUGS” (version 1.4.3; MRC Biostatistics Unit, Cambridge, UK). One of the major assumptions of the network meta-analysis was the consistency of two original pieces of evidence, which was dependent on whether the data, from direct and indirect evidence, could be combined on a basis of similarity^36^. To evaluate this assumption, we used the Bucher method^37^. We also assessed the consistency assumption in each closed loop of the network, and inconsistency was evaluated as disagreement between direct and indirect evidence with a closed loop^38^. The major computations were repeated by using a random effect model and a fixed effect model in order to implement a sensitivity analysis. R packages “metafor” and “combinat” were used in a consistency test, and “pcnetmeta” was used for the network diagram.

## Results

### Characteristics of eligible studies

**Figure 1:**
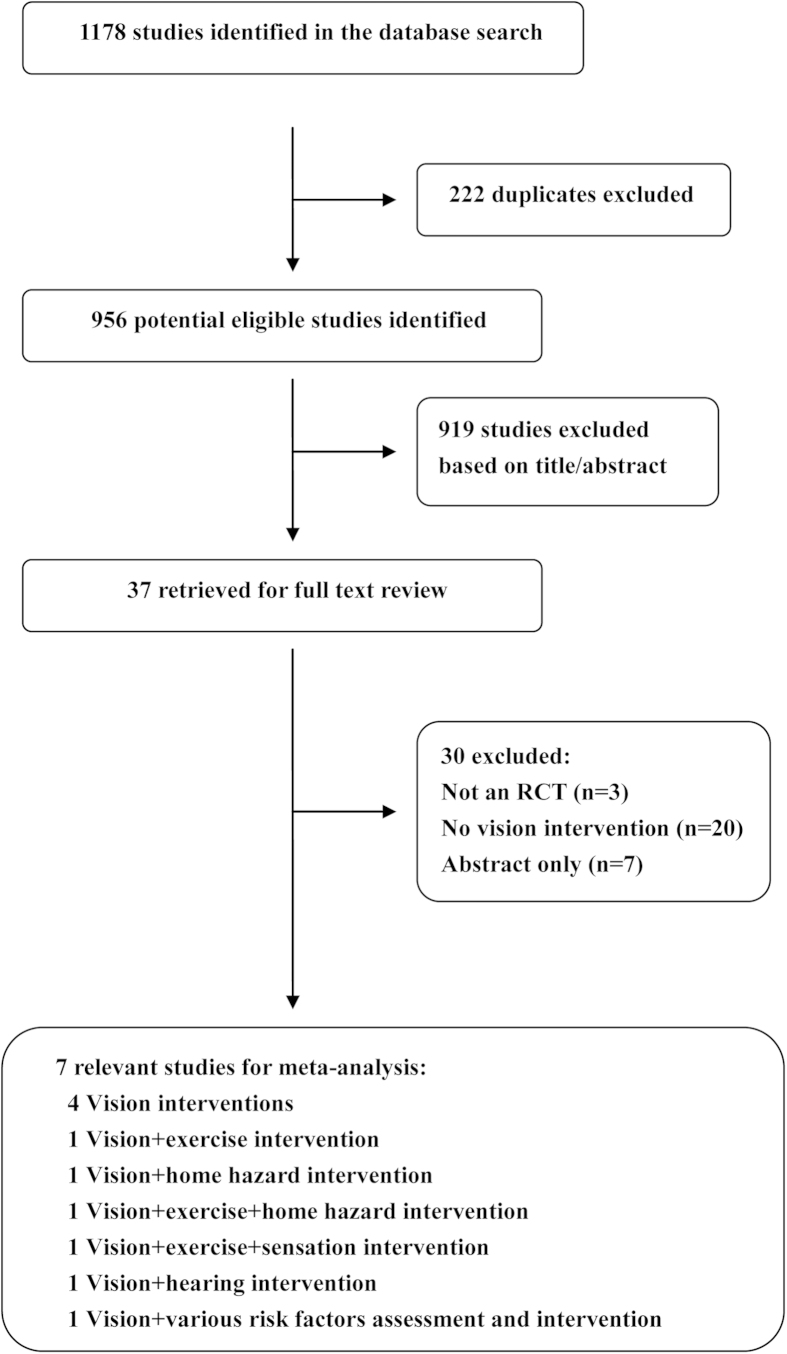
Flow chart of inclusion and exclusion in network meta-analysis. ‘*’expresses 7 RCTs incorporating 7 kinds of interventions in the analysis.

**Figure 2:**
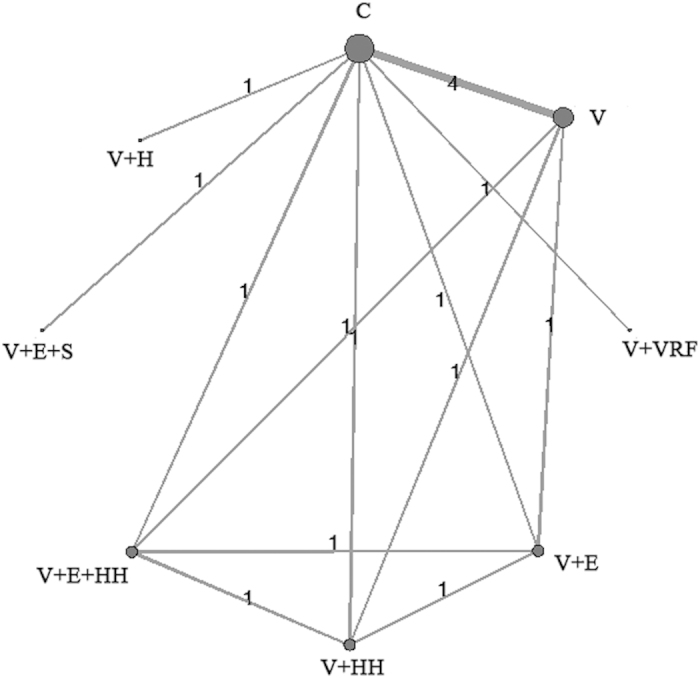
Diagrams of the network of eligible comparisons. Width of the lines represents the amount of studies for each pairwise intervention, and the size of node represents the number of participants respectively.

Through the literature search, we concluded 1178 records, and 37 eligible full text articles remained after screening titles and abstracts. Eventually, we included seven studies^13^,^24^,^25^,^26^,^27^,^28^,^29^, after the exclusion of 30 articles, comprising 2723 participants 65 years of age and over (median age ranging from 76 to 84 years), and were received one regimen among eight (Figs. 1 and 2). The average follow-up period ranged from 12 to 18 months, with most being 12 months. Seven studies were included in our network meta-analysis and the numbers of falls were used for analysis. All studies used no intervention as the control group.

### Falls outcome comparison

Fall outcomes were compared directly and showed in Table 2, indicating that V and V + VRF were better than C for lessening the incidence of older falls in this study. V + H produced more fall events than C.

### Outcomes of network meta-analysis

Based on a random effects model (Table 3 and Supplementary Table 1), the analysis outcomes of network meta-analysis usually had better conservative estimates and goodness of fit than the fixed effect model^34^. When comparing the falling incidence, all of the 95%CIs contained 1, indicating no statistical significance. From the rank rating, we can see a general trend in which the most effective intervention was V + VRF, the second was the V + E, and the third was V + E + HH. V was ranked second from the bottom. The last one was V + H. In addition, when we compared the incidence of fracture after falling, all of the 95%CIs contained 1, indicating no statistical significance (C, V, V + VRF were included in the comparison, because only four reports referred to the incidence of fracture). From the rank rating, the most effective intervention was V + VRF, the second was V. The last one was C. Furthermore, we utilized the Bayesian framework of the random effect model to rank the interventions and evaluate the cumulative probabilities of being the best intervention (Supplementary Table 2). An inconsistency factor for every closed loop was assessed when there was discrepancy between two source estimates. The 95%CI of each loop was compatible with zero, indicating the results between indirect and direct meta-analysis were not significantly different, and the consistency was relatively good.

We performed a sensitive analysis through comparing random and fixed effects models (Supplementary Table 3). Fortunately, the outcomes of the two models were consistent. The results were close, with no significant difference, indicating that the data was stable.

## Discussion

Conventional direct meta-analysis combines studies with the same pair of comparators. It was hard to integrate information on the relative efficacy of all regimens for the same indication. The question was resolved by network meta-analysis synthesizing direct and indirect evidence from trails^39^,^40^,^41^. Consequently, a network meta-analysis was used to compare and evaluate vision alone and vision plus other different interventions to increase a range of safety practices to prevent falls in older adults. In total, 7 RCTs, with a total of 2723 elderly participants with eight regimens, were included in analysis. Our objective is to synthesize evidence for vision intervention to prevent falls among older people, and find a more effective intervention combination.

A multidisciplinary review^42^ showed that visual impairment is an underrepresented area of research for falls among older adults. The authors of the review encourage further attention for the impact of visual deficits on fall risk. In our rank rating of interventions for lowering of falling incidence among the elderly, the most effective is V + VRF, followed by V + E, then V + E + HH. V was ranked second from the bottom, above V + H. The top three groups all are compound intervention groups, and included V and E. However, the rank rating of V was close to the bottom. A previous RCT found that only 5% of people with screen-detected vision problems received treatment, and improving vision might lead to changes in behavior that increase exposure to fall-prone situations^29^. On the other hand, vision related interventions for preventing falls have mainly focused on correcting central visual impairment, a study suggests that targeting both central and peripheral components may be necessary to effectively reduce rates of falls and falls with injury related to vision loss^43^. Consequently, it is not surprising that V alone is ineffective. From the analysis of falling incidence, we conclude that compound interventions are better than V alone. It is generally known that falling of the elderly is usually caused by various factors, so various risk factor interventions may be more effective than a single intervention.

In terms of ophthalmology, although 7 RCT studies all included V, the types of V were different. Among 7 publications, 5 studies recommended participants to an eye care specialist if vision did not reach a certain standard, and 2 studies used expedited cataract surgery as the V. According to the participant’s condition, eye care specialists will implement different Vs, such as changing new glasses or performing ophthalmological surgery. Visual acuity was improved through the different V methods, but insufficient correction by some interventions may influence the incidence of falling among older people.

There are some limitations to this network meta-analysis. Due to the small numbers of studies, we were not able to explore the impact of allocation concealment, blinding, and percentage follow-up separately. Fortunately, re-running the network meta-analysis, using data from RCTs, only had minimal impact on the results. The precision of results in our network meta-analysis might be affected by the follow-up time, in that the follow-up time in the 7 RCTs was not entirely consistent, and the different intensities of exposure to risk factors may impact falling incidence. However, the RCT is widely regarded as the gold standard for evaluating health care intervention. The RCT allows determination of a difference in outcomes that can be directly attributed to a difference in intervention and not to another factor.

We acknowledge that a meta-analysis of 7 studies may seem inadequate. However, we report our findings because we believe they contribute to the literature for several reasons. When definitive and large trials have not been performed to evaluate vision and other factors on the incidence of falls, a network meta-analysis of all available trials could help resolve some important issues, reducing the need for large, costly new trials. In this sense, our network meta-analysis provides more precise estimates of effectiveness and helps compare directly and indirectly the effects of vision intervention and vision plus other interventions in preventing falls by older people.

In conclusion, V + VRF proves to be more effective than other V combination interventions in preventing falls in elderly people. A single V appears less effective in our network meta-analysis.

## Additional Information

**How to cite this article**: Zhang, X.-Y. *et al.* Vision and Relevant Risk Factor Interventions for Preventing Falls among Older People: A Network Meta-analysis.. *Sci. Rep.* **5**, 10559; doi: 10.1038/srep10559 (2015).

## Supplementary Material

Supplementary Information

## Figures and Tables

**Table 1:** Results of Assessment for Bias in Included Trials.

	**Random sequence generation**	**Allocation concealment**	**Blinding**	**Incomplete outcome data**	**Selective outcome reporting**	**Other potential bias**
McMurd (2000)	unclear	unclear	unclear	adequate	no	yes
Harwood (2005)	adequate	adequate	inadequate	adequate	no	yes
Foss (2006)	adequate	adequate	inadequate	adequate	no	yes
Cumming (2007)	adequate	unclear	unclear	adequate	no	yes
Day (2002)	adequate	unclear	adequate	inadequate	no	yes
Lord (2005)	adequate	adequate	adequate	adequate	no	no
Grue (2008)	inadequate	unclear	unclear	inadequate	no	yes

Abbreviations: C = control group; V = vision intervention; V + VRF = vision plus various risk factor interventions; V + E = vision plus exercise interventions; V + HH = vision plus home hazard interventions; V + E + HH = vision plus exercise plus home hazard interventions; V + E + S = vision plus exercise plus sensation interventions; V + H = vision plus hearing interventions.

**Table 2:** Direct comparisons in traditional meta-analysis.

	**OR**	**95%CI**	**P**	**I^2^**
V vs. C	0.39	0.07-0.70	0.017	40.18
V + H vs. C	1.70	1.20-2.50	0.004	
V + E + S vs. C	1.01	0.68-1.48	0.973	
V + VRF vs. C	0.37	0.17-0.80	0.011	

Abbreviations: CI = confidence interval; OR = odds ratio; V = vision intervention; C = control group; V + H = vision plus hearing interventions; V + E + S = vision plus exercise plus sensation interventions; V + VRF = vision plus various risk factor interventions. One eight-arm study was excluded from the analysis. OR: odds ratio; CI: confidence interval.

**Table 3:** Result of network meta-analysis on falling incidence of the seven intervention regimens and control group.
