# Supplementary material for: Vision and Relevant Risk Factor Interventions for Preventing Falls among Older People: A Network Meta-analysis
Source: Sci Rep. 2015 May 28;5:10559. doi: 10.1038/srep10559 (PMC4447164; doi:10.1038/srep10559)
Supplement: Supplementary Information [file srep10559-s1.doc]

Vision and Relevant Risk Factor Interventions for Preventing Falls among Older People: A Network Meta-analysis

Supplement Table 1

| \| closed loops \| IF with 95%CI \| \| --- \| --- \| \| control.V+E.V+HH \| 0.00[-1.11,1.11] \| \| control.V+E.V+E+HH \| 0.08[-0.95,1.12] \| \| control.V+E.V \| 0.17[-0.80,1.15] \| \| control.V+HH.V \| 0.17[-0.76,1.10] \| \| control.V+E+HH.V \| 0.17[-0.80,1.15] \| \| V+E.V+HH.V \| 0.00[-0.12,1.12] \| \| V+E.V+E+HH.V \| 0.08[-0.96,1.13] \|   V:vision intervention |
| --- | --- | --- | --- | --- | --- | --- | --- | --- | --- | --- | --- | --- | --- | --- | --- | --- |
| E:exercise intervention |
| HH: home hazard intervention |
| Supplement Table 2   \|  \|  \| **falls incidence** \| \| --- \| --- \| --- \| \| **Rank** \| control \| 4.78(2.00-7.00) \| \|  \| V \| 6.17(3.00-8.00) \| \|  \| V+VRF \| 2.46(1.00-8.00) \| \|  \| V+E \| 2.97(1.00-7.00) \| \|  \| V+HH \| 4.57(1.00-8.00) \| \|  \| V+E+HH \| 3.01(1.00-7.00) \| \|  \| V+E+S \| 4.82(1.00-8.00) \| \|  \| V+H \| 7.23(2.00-8.00) \| \| **Best** \| control \| 0.01(0.00-0.00) \| \|  \| V \| 0.00(0.00-0.00) \| \|  \| V+VRF \| 0.49(0.00-1.00) \| \|  \| V+E \| 0.20(0.00-1.00) \| \|  \| V+HH \| 0.04(0.00-1.00) \| \|  \| V+E+HH \| 0.18(0.00-1.00) \| \|  \| V+E+S \| 0.07(0.00-1.00) \| \|  \| V+H \| 0.01(0.00-0.00) \| \|  \|  \|  \| |

| V:vision intervention | | | |  |
| --- | --- | --- | --- | --- |
| E:exercise intervention | | | |  |
| HH:home hazard intervention | | | |  |
| VRF:various risk factors intervention | | | |  |
| S:sensation intervention | | | |  |
| H:hearing intervention  Supplement Table 3 | | | |  |
|  | totresdev | pD | DIC | |
| **fixed** | 21.5 | 14.1 | 127.4 | |
| **random** | 16.95 | 16.3 | 125 | |
